# Supplementary material for: Shared Treg dysregulation underlies psoriasis vulgaris and atopic dermatitis through the PHF19-PRC2 epigenetic axis
Source: Front Immunol. 2026 Aug 20;17:1932788. doi: 10.3389/fimmu.2026.1932788 (PMC13538097; doi:10.3389/fimmu.2026.1932788)
Supplement: Supplementary file 3 [file DataSheet3.zip › Additional files/Additional file 6/12.PPP2R3C.forest.pdf]

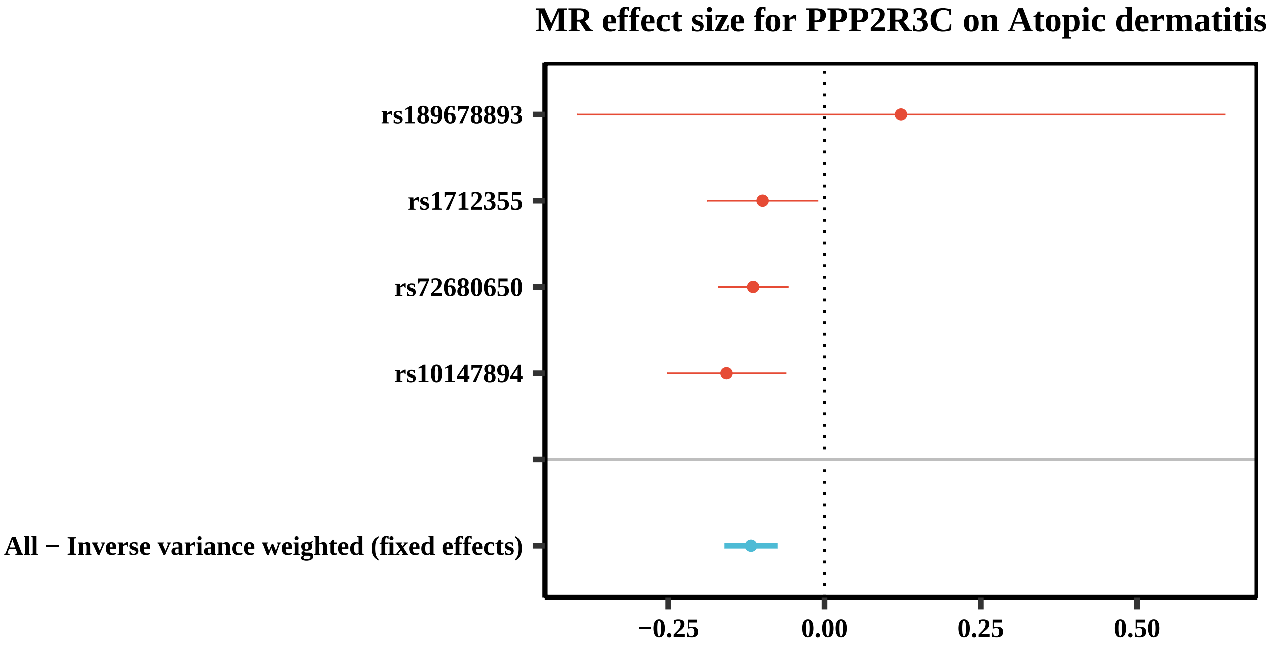
MR effect size for PPP2R3C on Atopic dermatitis
rs189678893
rs1712355
rs72680650
rs10147894
All − Inverse variance weighted (fixed effects)
−0.25
0.00
0.25
0.50
